# Secular trends and evaluation of complex interventions: the rising tide phenomenon

**DOI:** 10.1136/bmjqs-2015-004372

**Published:** 2015-10-06

**Authors:** Yen-Fu Chen, Karla Hemming, Andrew J Stevens, Richard J Lilford

**Affiliations:** 1 Warwick Centre for Applied Health Research & Delivery, Division of Health Sciences, Warwick Medical School, University of Warwick, Coventry, UK; 2 School of Health and Population Sciences, University of Birmingham, Birmingham, UK

**Keywords:** Evaluation methodology, Cluster trials, Quality improvement, Health services research, Randomised controlled trial

## Abstract

Evaluations of service delivery interventions with contemporaneous controls often yield null results, even when the intervention appeared promising in advance. There can be many reasons for null results. In this paper we introduce the concept of a ‘rising tide’ phenomenon being a possible explanation of null results. We note that evaluations of service delivery interventions often occur when awareness of the problems they intend to address is already heightened, and pressure to tackle them is mounting throughout a health system. An evaluation may therefore take place in a setting where the system as a whole is improving – where there is a pronounced temporal trend or a ‘rising tide causing all vessels to rise’. As a consequence, control sites in an intervention study will improve. This reduces the difference between intervention and control sites and predisposes the study to a null result, leading to the conclusion that the intervention has no effect. We discuss how a rising tide may be distinguished from other causes of improvement in both control and intervention groups, and give examples where the rising tide provides a convincing explanation of such a finding. We offer recommendations for interpretation of research findings where improvements in the intervention group are matched by improvements in the control group. Understanding the rising tide phenomenon is important for a more nuanced interpretation of null results arising in the context of system-wide improvement. Recognition that a rising tide may have predisposed to a null result in one health system cautions against generalising the result to another health system where strong secular trends are absent.

## Introduction

Interventions to combat health service delivery problems (such as hospital-acquired infections) are often developed in response to a heightened public awareness and mounting pressure to tackle them. Under these circumstances, a groundswell of public and professional opinion may be the stimulus for both a spontaneous change across a health system and formal evaluations of particular interventions within that system. Service delivery interventions are often complex in the sense that they are made up of a number of components, many of which may not be novel and which, unlike pharmaceuticals, are not restricted by licensing requirements. The result is that interventions of various types diffuse into widespread practice in an uncontrolled way while evaluation studies are under way. For example, concerns over hospital-acquired infections may lead hospitals across the system to adopt methods to improve hand hygiene and these same concerns may also stimulate formal research studies to evaluate specific interventions with the same aim. Insofar as these various interventions are effective, they produce a positive secular trend. We shall use the metaphor of a ‘rising tide’ as a short hand for such a secular trend that is contemporaneous with the evaluation of an intervention. Such a rising tide may obscure the measured effect of an intervention in a study with contemporaneous controls. Appreciating the possibility of a rising tide offers additional insight for interpreting null results where both control and intervention sites have improved.

This paper aims to illustrate the rising tide phenomenon in which this might explain a null result where both intervention and contemporaneous control sites have improved. We discuss evidence that may help distinguish between a rising tide and alternative explanations for the null result, and illustrate this approach with examples.

## Temporal trends versus other explanations for improvement across intervention and control sites

The possibility of a ‘rising tide’ explanation arises when a controlled study with baseline measurement(s) yields a null result in which there has been improvement across both intervention and control sites.

Various criteria can be put forward to help distinguish a rising tide from other explanations for such simultaneous improvement—we offer these in the spirit of Bradford Hill's famous criteria for cause–effect explanations in clinical research.^1^ Leaving aside the play of chance (which will have been calibrated statistically), the probability of the rising tide explanation increases in proportion to evidence for the existence of a rising tide and declines in proportion to evidence supporting rival explanations.

Evidence for a rising tide, from strongest to weakest, consists of the following:

1. Data showing that improvement similar to that in study sites occurred across the healthcare system as a whole. Such external data may be derived from regular population surveys, national registries, or routine administrative databases and provide direct evidence of a positive secular trend.
2. Data showing that the intervention and control sites within a study had started to improve before the intervention came on stream, so that it was a continuation of a trend in both intervention and control sites.
3. Qualitative evidence, say in the form of interviews with staff, showing strong motivation to improve practices in both intervention and control sites.
4. Circumstantial evidence in the form of press articles, government reports, and/or documents from national societies showing that the topic was one of pervading concern.

Contamination is the most immediate rival explanation for simultaneous improvement in both intervention and control groups. Contamination is used here in the standard epidemiological sense that control sites become aware of the intervention and replicate it to some degree,^2^ ^3^ thereby diluting the estimated effect; the direction of effect is from intervention sites to control sites *within* a study, biasing results towards the null. The intervention ‘leaks’ from intervention to control sites and must follow allocation to intervention and control conditions. A rising tide, by contrast, impacts on all sites in a system, irrespective of whether they are or are not included in the study and it may precede allocation of intervention and control groups. Contamination should be suspected when it can be demonstrated that participants in the control group were exposed to elements of the intervention that had ‘spilled over’ from the intervention group *within* the study (rather than from outside).

It is possible for other sources of bias (see table 1) to create or exaggerate the appearance of an improvement in the control group or even to create the illusion of improvement in the intervention group, when in fact it was mainly or only the control group that had improved. Bias could arise, for example, if there was higher dropout from control than intervention sites or if controls were subject to selection bias.

**Table 1:** Issues to be considered for assessing a rising tide phenomenon and results of assessment for the four case studies*

	SPI2	Critical pathways	EQHIV	MERIT
*Positive evidence*				
Direct evidence				
Improvement in process and/or outcome measures observed in external sites:	Yes	Yes	Yes	Yes
Timing: before or during evaluation study	Before and during	Before and during	Before	During
System-wide or specific external site(s)	System-wide	Specific external sites	System-wide	System-wide (but 30% participation)
Qualitative evidence showing behaviour changes driven by external factors in both study groups	Yes	Yes	Yes	Yes
Suggestive evidence				
Baseline measures better than expected, or already showing high standards or improving trend	Yes	Yes	Yes	Yes
Circumstantial evidence				
Heightened awareness of the problems	Yes	Yes	Yes	Yes
*Negative evidence*†				
Contamination within study	No	No	Unlikely	Unlikely
Other potential sources of biases‡	Not apparent	Not apparent	Attrition bias cannot be ruled out	Not apparent

*Improvement in process and/or outcome measures were observed in both intervention and control groups in these studies during the evaluation period.

†Factors of which the impact on study findings could resemble a rising tide phenomenon.

‡Including selection bias (eg, control group being a selective sample of highly motivated units or having more headroom for improvement), bias in outcome assessment (eg, changes in methods of data collection or coding over time) and attrition bias (eg, poor-performing units dropping out and being excluded from analysis).

## Examples of a putative rising tide phenomenon

In this section we provide four examples from published literature in which a rising tide phenomenon may be suspected. We briefly describe the key features of these studies and illustrate how the criteria mentioned above and listed in table 1 can be applied to help inform a judgement on the likelihood of a rising tide explanation versus alternative explanations.

**Figure 1:**
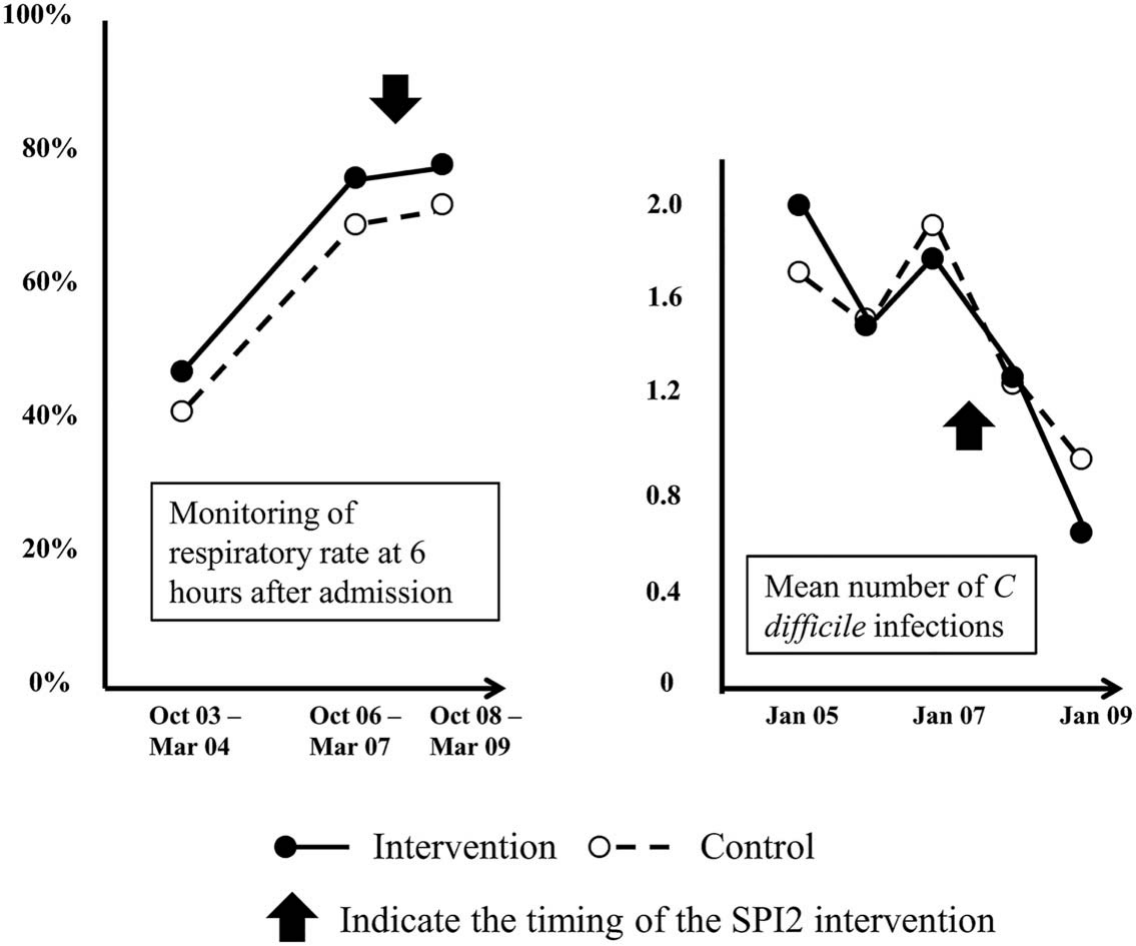
Key characteristics and findings of the Safer Patients Initiatives phase 2 (SPI2). This controlled before-and-after study evaluated a multi-component intervention using an organisation-wide approach to improve patient safety. The key components included interventions to facilitate generic improvement in the hospital system to reduce adverse events (such as building a good leadership to support a culture of safety as well as interventions targeting specific clinical processes that carry a relatively high risk of adverse events (such as procedures aiming to enhance infection control). Various outcomes were measured, including staff morale, culture and opinion, the quality of acute medical care and perioperative care, use of consumables for hand hygiene, adverse events and hospital mortality in older patients with acute respiratory disease, intensive care unit mortality, infection rates associated with healthcare and patients satisfaction. SPI2 was preceded by a pilot phase (SPI1) that provides data on the pre-implementation phase for certain end-points, including the two outcomes illustrated here.

Our first example, the Safer Patients Initiative phase 2 (SPI2) study, was a controlled before-and-after evaluation of a multicomponent hospital clinical safety programme.^4^ ^5^ Many dimensions of quality measured in the study improved over the intervention period (spanning from March 2007 to September 2009), but did so equally in both intervention and control groups (figure 1). One of the targets of the intervention was to improve recognition of deteriorating patients in general wards, and the quality of nursing observations (as judged from masked review of the notes) improved markedly and statistically significantly over the study period, but no difference was observed in the rate of improvement across intervention and control sites. Likewise, use of hand washing materials improved over time but at a similar pace across sites. There was evidence of improving standards of monitoring in control and intervention sites (which started before the intervention was implemented).^5^ There were widespread national initiatives to improve the standard of monitoring on the wards,^6^ and external evidence showed increased use of hand wash materials and reduced infection rates across the whole of England over the study period.^7^ ^8^ Contamination, in the sense described above, is very unlikely—controls were recruited retrospectively and data were obtained retrospectively from case notes and routine data. For these reasons, the controls would not have been aware that they were controls at the time of intervention. This is an example of an arguably unusual situation where there is specific strength in retrospective selection of control sites.

**Figure 2:**
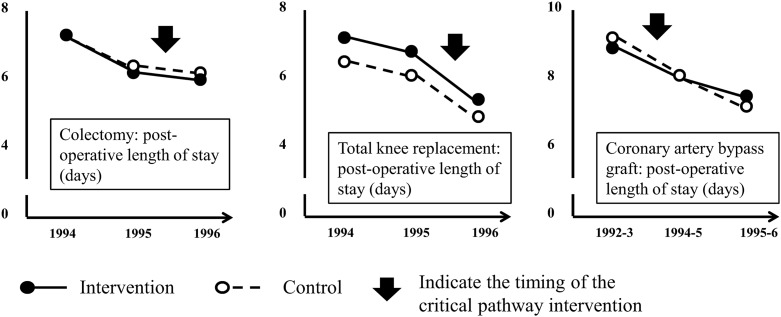
Key characteristics and findings of the Critical Pathways Intervention. This controlled before-and-after study compared the effects of a quality improvement initiative utilising critical pathway framework on post-operative length of stay in a large teaching hospital with concurrent data from 2-3 similar neighbouring hospitals without the intervention. Multidisciplinary teams identified critical steps in the care process and specified required actions and desirable outcomes for each step. Patients entered into critical pathways were monitored and various methods of benchmarking and feedback were used in pathway management. The primary outcome was post-operative length of stay. Hospital charges and other process and clinical outcomes were also examined.

The Critical Pathway Program was an initiative started in 1993 in the Brigham and Women's Hospital (Boston, USA) to improve efficiency in service delivery for high-cost, high-volume surgical procedures.^9^ A controlled before-and-after evaluation for its application in colectomy, total knee replacement, and coronary artery bypass graft surgery showed substantial and statistically significant reductions in the average length of hospital stay for all three procedures in both intervention and control sites. Data from the 2 years before intervention suggested that length of stay had started to decline in both intervention and control hospitals before the intervention was initiated in the former (figure 2), and external nation-wide US data showed a continuous decrease in average length of hospital admission spanning the period of the Critical Pathways Intervention, from 9.1 days in 1990 to 7.8 days in 1995 and 7.0 days in 1999.^10^ Staff interviews at control hospitals provided evidence that competitive pressure, rather than contamination, had triggered efforts to reduce length of stay and improve efficiency.

**Figure 3:**
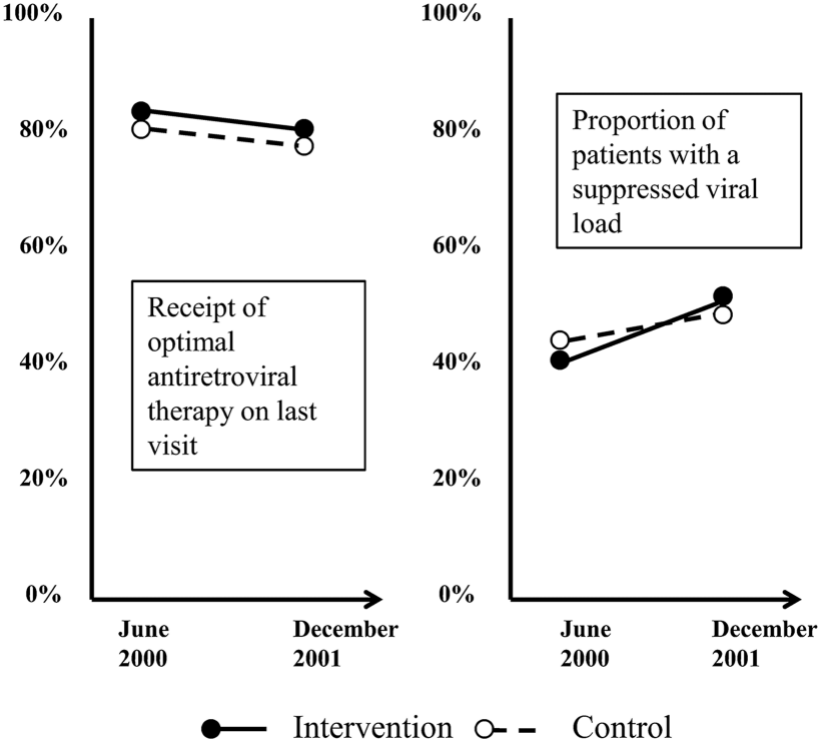
Key characteristics and findings of the EQHIV study. This controlled before-and-after study evaluated the effectiveness of the ‘Breakthrough Series’, a multi-institutional quality improvement collaborative led by Institute for Healthcare Improvement, on improving the quality of case for clinics treating HIV infected patients. The 16-month intervention involved a series of meetings (learning sessions) covering the theory and practice of quality improvement in the intervention clinics and sharing ideas and progress between them. The primary outcome measures were rates of optimal antiretroviral therapy use and control of HIV viral load.

EQHIV was a controlled before-and-after study evaluating the effectiveness of a suite of interventions to improve the quality of care in clinics treating HIV-infected patients.^11^ Among the outcome measures, the proportion of patients whose viral load was adequately suppressed increased significantly within each group—by a greater extent in the intervention group (11%; from 41% to 52%) than the control group (6%; from 44% to 50%). However, the between-group difference was not statistically significant (p=0.18). Compliance with a prescription guideline was already high at baseline and did not increase further in either group after the intervention (figure 3). National data from the HIV Cost and Services Utilization Study showed that EQHIV was preceded by significant improvement in care of HIV-infected adults.^12^ Interview of clinical directors in study sites suggested minimal contamination, as those in control sites reported many fewer quality improvement initiatives compared with intervention sites. However, attrition bias cannot be ruled out, as only 63% (25/40) of selected control sites provided sufficient data to be included in analysis.

**Figure 4:**
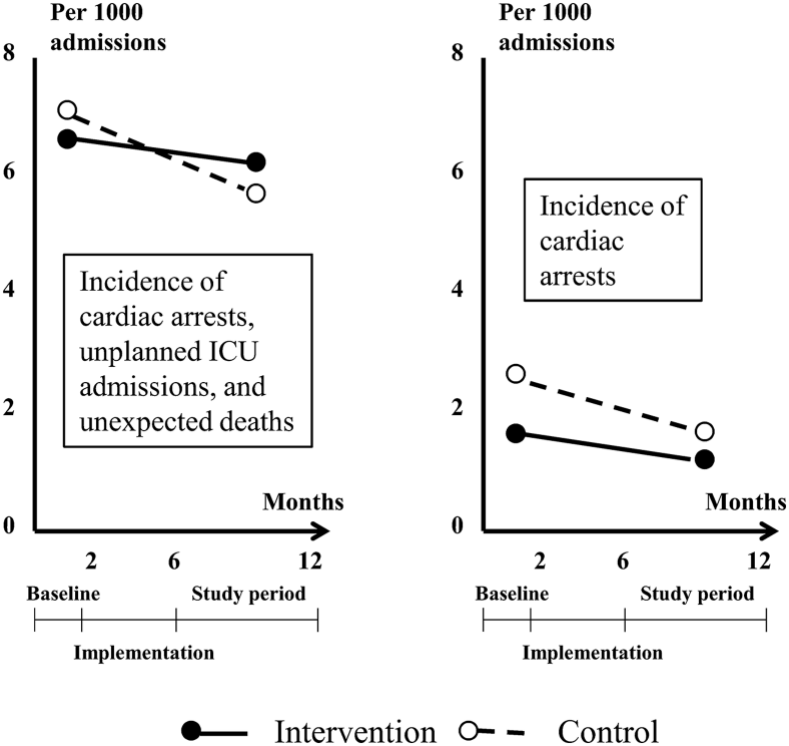
Key characteristics and findings of the MERIT study. This cluster randomised controlled trial (RCT) investigated the effectiveness of introducing a medical emergency team (MET) that could be summoned when non-terminal hospital patients showed signs of physiological instability and deterioration. Staff in the intervention hospitals were trained over 4-months to identify and respond to patients requiring the attention of the team. The primary outcome was the combined incidence of cardiac arrests without a pre-existing not-for-resuscitation order. unplanned intensive care unit admissions and unexpected deaths measured before and after implementation.

MERIT was a cluster randomised controlled trial of the effectiveness of emergency teams for deteriorating non-terminal hospital patients in reducing the combined outcome of cardiac arrests without a pre-existing not-for-resuscitation order, unplanned intensive care unit admissions, and unexpected deaths.^13^ Before the intervention began, the incidence of the outcome appeared to have already improved from 26 per 1000 admissions estimated in a previous study,^14^ to 6.6 and 7.1 per 1000 admissions observed at baseline for intervention and control hospitals, respectively.^13^ ^15^ Further improvement was observed in both intervention and control groups after the intervention, with no significant difference between groups (reduction of 0.39 vs 1.41 per 1000 admission for intervention vs control, p=0.30). Similar findings were observed for secondary outcomes (figure 4). External evidence of a secular trend and widespread adoption of medical emergency teams comes from a national registry in which about 30% of all intensive care units provided relevant data.^16^ The risk of contamination was minimised by agreement of control hospitals not to publicise the intervention internally and not to change the operation of their cardiac arrest team during the study period.

In summary, there is evidence for a secular trend in all four cases. The case for a rising tide is strongest for SPI2 and MERIT where data from both within and outside the study pointing towards a system-wide secular trend and evidence for alternative explanations can largely be ruled out. For the remaining cases, there is some uncertainty, mainly arising from lack of evidence to eliminate alternative explanations. On the whole, the evidence (summarised in table 1) indicates that a secular trend is likely to have contributed to the null results observed in all four studies.

## Discussion

### What causes a rising tide?

Widespread concern about an issue such as hospital-acquired infection or medication error may motivate multiple changes throughout a system that includes, but is not limited to, sites involved in a research study. Exactly how these changes are propagated is a large subject for study, save to say that human behaviour is strongly influenced by prevailing social attitudes and practice.^17^ Two points can be made about the phenomenon of the spread of behaviour in a community of practitioners:

1. It is not necessary to postulate that the way in which organisations respond to social ‘forces’ is the same everywhere. Services may be improved in a number of separate ways,^18^ and improvement across the system might arise from intervention and non-intervention sites adopting the same practices, separate practices of similar efficiency, or a mixture of similar and different practices. An analogy of the multifarious ways that social forces may cause a rising tide is shown in box 1. The intervention group is also subject to the rising tide. The measured intervention effect in a study inclines towards the null if the effect of the intervention attenuates with increasing ‘dose’ and/or if the headroom for further improvement is consumed.
2. A rising tide can only produce a null result if there is at least some temporal overlap between widespread promulgation of the interventions and the evaluation of a particular intervention. However, it is not necessary for system-wide change and research to start simultaneously and such timing is unlikely given the lag in establishing research projects. Indeed improvement originated before the study got underway in three of the four above examples (table 1).

Box 1 **Evolutionary analogy of the rising tide phenomenon**

A naturalist studying desert fauna may notice that they have certain features in common. It might first be observed that mice are sand-coloured. Then that the snakes, lizards and small birds are all of similar hue. The naturalist may observe that this would increase their efficiency as both prey and/or predator (both, in the case of the snake). Under the same external influence (changes in physical environment), organisms evolve similar outcomes (a sandy colour) by different means (distinct biochemical pathways). So it is that, under the same primary driver (changes in the social environment), organisations evolve similar outcomes (fewer infections) by different means (such as promoting hygienic practices and screening all patients for resistant bacteria on admission).

### Detecting a rising tide explanation

A rising tide phenomenon is, in essence, a pronounced secular trend created by social responses to a particular issue which has gained widespread attention. While it is impossible to find incontrovertible proof of a rising tide explanation, we have assembled a set of criteria that should be taken into account in the interpretation of controlled evaluations that have generated a null result associated with similar improvement in both intervention and control groups (table 1). Indeed, possible influence of a secular trend was mentioned or alluded to by the authors of all the case examples we presented here. It must be emphasised that a rising tide does not preclude a positive result. The intervention may augment widespread contemporaneous change because the intervention is different (at least in part), and/or administered with different intensity. For instance, in a controlled evaluation of a team training programme for operating room personnel, a statistically significant reduction in risk-adjusted surgical mortality rate was observed, despite a 7% decrease in annual mortality rate in the control group.^19^ A tide may also recede, in which case a successful intervention may be one that arrests decline, but this would be manifested as a positive result, not a null result.

### Rising tide in applied health research

Situations analogous to the rising tide phenomenon can occur in a variety of applied health research—for example, in a trial of screening for prostate cancer, where a substantial proportion of the population (and hence the control group) underwent screening.^20^ Likewise, an educational package for general practitioners to apply more intensive antidepressant treatment was evaluated at a time when the idea was already getting national publicity.^21^ Many other examples can be found in the realm of service delivery interventions.^22^ ^23^ Most recently, the rising tide phenomenon is likely to have contributed to the null findings from two independent analyses of the effect of participation in the American College of Surgeons National Surgical Quality Improvement Program (ACS NSQIP), where mortality and certain other outcomes improved in both intervention and control groups,^24^ ^25^ and in the English Matching Michigan study where the rate of decline in central venous catheter bloodstream infection following the introduction of the intervention in intensive care units was not significantly different from a concurrent temporal trend.^23^

### Does it matter?

We have described a set of criteria to help decide whether a null result in the face of improving outcomes can be attributed to a rising tide (table 1). One subset of criteria concerns a convincing alternative explanation, particularly contamination. It could be argued that a null result needs no further explanation once one is satisfied that it has been measured with sufficient precision and decided that an alternative explanation, such as contamination, can be excluded. Contrary arguments are now given based on two rather distinct philosophical traditions.

1. We draw attention to a distinction made by Schwartz and Lellouch^26^ between pragmatic and explanatory motivations for a study. The former consists of generating information to inform a particular prespecified decision, and the second consists of generating an understanding of causal mechanisms. A null result in the face of a rising tide fulfils the first, but not the second, requirement. It fulfils the first (pragmatic) requirement because, if a study designed (and powered) around the decision makers’ requirements is assumed, an incremental effect size sufficient to justify the marginal costs of the intervention is excluded. However, the second (explanatory) requirement is unsatisfied, since it does not indicate what the effect of the study intervention would be in a system that was not experiencing a positive temporal trend. In such a system, the intervention would not be ‘competing’ with other positive changes in the system.
2. The second philosophical argument turns on the idea that it is wrong to make decisions based solely on a statistical convention,^27^ as pointed out in Sir Bradford Hill's famous lecture.^1^ To put this another way, data should contribute to an understanding of causal mechanisms (theory), and the rising tide may help explain why an intervention that was expected to prove effective yielded a null result.

### Recommendations for future practice

Having discussed the idea of a secular trend phenomenon, we propose here some options that can be considered alongside established guidelines^28–31^ during the design of evaluation studies for service and policy interventions in order to facilitate correct interpretation of study findings.

1. In many cases, at least some of the study end points will be available from routine administrative databases or independent surveys regularly carried out nationally. This will allow verification of whether a change observed in the evaluation study is associated with the study participation itself or is similarly observed elsewhere outside the study, thereby providing strong evidence of a secular trend, at least as far as shared end points are concerned. This was the case in the SPI2 study.
2. Qualitative data may provide evidence to explain study results;^5^ ^32^ in the case of SPI2, behaviour change was driven by factors in the external environment in both intervention and control sites.
3. Obtaining multiple measurements spanning the pre- and post-intervention period —that is, a controlled interrupted time series.^29^ Multiple observations before the intervention phase may provide evidence of long-term secular trends in both control and intervention groups.^33^ ^34^
4. Prior to the start of data collection, the sample size can be adjusted to take account of secular trends when these are expected. Such analysis can be used to assess the feasibility and value of an evaluation study before it is commissioned, or to inform a decision on whether to extend an ongoing study by increasing its size or to terminate it on grounds of ‘futility’.^35^ ^36^
5. Considering designs that allow temporal effects to be modelled. One example is a step wedge design,^37^ which uses randomisation as a method to determine the order in which centres on a waiting list receive the intervention. It has many logistical, political and even ethical advantages over a parallel design,^28^ ^38^ ^39^ and (given a sufficiently large sample) also allows the intervention effect, general temporal effects, and any effect on the intervention at the time it was introduced to be modelled.

## Conclusion

Social pressure that triggers the development and evaluation of a service delivery intervention may at the same time drive spontaneous, widespread changes in a health system leading to improvement across the board, which we describe here as a rising tide. Controlled evaluation studies undertaken amidst a rising tide may yield a null result because incremental effects are similar between intervention and non-intervention sites. Recognition of a rising tide is important because, while the null result demonstrates pragmatically that the intervention does not produce sufficient incremental benefit in this particular scenario, it leaves open the possibility that the intervention could work in a different scenario where a rising tide is absent.

In this paper we offer four case studies of evaluations of complex interventions to illustrate a rising tide phenomenon, and suggest a framework to assess evidence either supporting or refuting its presence. Our aim is to raise awareness of the phenomenon and of its potential implications in the design and interpretation of evaluation studies. Further work to gather empirical evidence on the occurrence of such a phenomenon and to develop methods to delineate its impact from other bias is the next step. This in turn will provide guidance for health services researchers and decision makers on the optimal actions to take in the face of a rising tide.
